# Editorial: Protecting Our Crops - Approaches for Plant Parasitic Nematode Control

**DOI:** 10.3389/fpls.2021.726057

**Published:** 2021-09-01

**Authors:** Juan E. Palomares-Rius, Koichi Hasegawa, Shahid Siddique, Claudia S. L. Vicente

**Affiliations:** ^1^Institute for Sustainable Agriculture-Consejo Superior de Investigaciones Científicas (CSIC), Córdoba, Spain; ^2^Department of Environmental Biology, Chubu University, Kasugai, Japan; ^3^Department of Entomology and Nematology, University of California, Davis, Davis, CA, United States; ^4^MED-Mediterranean Institute for Agriculture, Environment and Development, Institute for Advanced Studies and Research, University of Évora, Évora, Portugal; ^5^INIAV, I.P. – Instituto Nacional de Investigação Agrária e Veterinária, Oeiras, Portugal

**Keywords:** plant-parasitic nematodes, plant-nematode interactions, nematode-microbe interactions, sustainable agriculture, biocontrol, breeding

In agricultural history, the Green Revolution generated by the development of breeding technology, chemical fertilizers, and pesticides has enabled mass production of agricultural crops and solved many (but not all) hunger problems around the world (Pingali, 2012). Plants make up about 80% of the food we consume, while about 40% of food crops are lost by agricultural pests, including plant nematodes (FAO, 2019). The world population in 2021 is ~7.8 billion and is estimated to reach 10 billion in 2050 (United Nations, 2019). The current proposition imposed on us is to develop methods to increase crop yield and quality while suppressing damage from pests and also reducing the impact on the natural environment. Plant-parasitic nematodes (PPNs) are one of the major constraints in agriculture. Damage caused by PPNs has been estimated from $US80 billion (Nicol et al., 2011) to $US157 billion per year (Abad et al., 2008). However, the full extent of nematode damage is likely underestimated as many growers, particularly in developing countries, are unaware of the presence of PPNs (Jones et al., 2013). This was assumed as nematodes are usually small-body-size, soil-borne pathogens, and the symptoms they cause are often non-specific (Jones et al., 2013). The damage caused by PPNs could be even worse in the future in the context of a growing world population under a Climate Change scenario and the removal or reduction in the use of some nematicides in many parts of the world. Set in the context of the 2020 International Year of Plant Health, this Research Topic “Protecting Our Crops - Approaches for Plant Parasitic Nematode Control” gives new insights into Integrative Approaches for Sustainable PPN Control. Many of the articles are excellent reviews of their specific topic, which could help in pointing out new research directions.

The knowledge of the geographical distribution, prevalence, and diversity of specific PPNs is a keystone point for further development of control measures in a region or country as well to prevent the dispersion of the specific pathogen in unaffected areas. This distribution could be influenced by Climate Change, which forecasts an increase in global temperature with important temperature and precipitation instability and could induce a northward migration/higher altitude shifts and an increase in the damage severity in areas of current distribution (Chakraborty et al., 2000). Climate Change could affect crops (e.g., plant defenses, growth, or period of sowing) as well as the Biological Control Agents (BCAs) associated with a specific nematode. Potato cyst nematodes (*Globodera pallida* and *G. rostochiensis*) are the most economically important parasitic nematodes of the potato (*Solanum tuberosum*), in many cases inducing important losses to farmers by reducing production or regulatory restrictions in their trade. These nematodes are challenging due to their survival strategy. Cysts produced by the female's cuticle hardening are highly durable, and viable eggs, protected inside the cysts, can survive for decades in the absence of a host (Turner, 1996). The data revealed about potato cyst nematodes in Kenya (Mburu et al.) and Portugal (Camacho et al.) showed two different scenarios with a widely spread predominant species (*G. rostochiensis*) in Kenya (Mburu et al.) and the wide presence of both species (*G. pallida* and *G. rostochiensis*) in Portugal (Camacho et al.) and differences in the use of resistance cultivars in these two countries. Additionally, the root-knot nematode (RKN) *Meloidogyne enterolobii* (syn. *M. mayaguensis*) has been considered as a global threat for tomato (*Solanum lycopersicum*) production due to the lack of known resistance in commercially accepted tomato varieties (*Mi*-genes does not control *M. enterolobii*) (Moens et al., 2009; Castillo and Castagnone-Sereno, 2020). This species has a broad host range and has been recently found in many countries worldwide in areas with a subtropical to tropical climate (Castillo and Castagnone-Sereno, 2020; Subbotin et al., 2021). Philbrick et al. review the current knowledge on geographic distribution, host range, population biology, control measures, and proposed future strategies to improve *M. enterolobii* control in the tomato. Growing resistant varieties is the most environmentally and economically friendly method to combat RKNs in the field (Seid et al., 2015), but in many cases exerts selection pressure on nematode populations, increasing the number of virulent individuals (Whitehead, 1991; Kaloshian et al., 1996; Hockland et al., 2012) and the need for accurate strategies for sustainable management of the nematode resistance in the field (Barbary et al., 2015). Gartner et al. provide an interesting review of the different resistance genes, their management, and the recent advances in potato genomics to develop molecular markers to aid selection during breeding. Cui et al. characterize the resistance to cereal cyst nematodes (*Heterodera aveane* and *H. filipjevi*), agronomic performance, and end-use quality parameters in four perennial wheat-*Thinopyrum intermedium* lines. Another approach in this research line is the use of alternative crops/cover crops or double-cropping. Double-cropping is defined as producing more than one crop on the same parcel of land in a single growing season, increasing biomass productivity, weed control, and supplying the increased demand for food and feed (Leslie et al., 2017). Rocha et al. studied the use of double-cropping to control one of the major soybean (*Glycine max*) pathogen problems, the soybean cyst nematode (SCN) (*Heterodera glycines*). Double-cropping soybean with wheat (a non-host) has the potential to suppress SCN field populations in comparison to fallow (Rocha et al.). Additionally, this system provides additional farm income. The evidence of these techniques is also evaluated by Rashidifard et al. by studying the crop rotation with sainfoin (*Onobrychis viciifolia*) to reduce *M. enterolobii* infection of maize under greenhouse conditions in a pilot approach. Their results showed that the rotation of sainfoin Esparsette/maize reduced *M. enterolobii* population density by 81 and 60% in the first and repeat experiments, respectively, followed by alfalfa (*Medicago sativa*) 54 and 43%, respectively (Rashidifard et al.).

Novel control methods for PPNs are needed in the context of sustainable agriculture using fewer inputs and being more respectful of the environment and the health of consumers and applicators. Many nematicides are in decline in terms of use or are being banned because of adverse environmental impacts. In this sense, several approaches are pointed for the use of specific plant compounds for the control of PPNs. In this Research Topic, Desmedt et al. have reviewed the metabolites used by plants to defend themselves against PPNs. Metabolites are reviewed by chemical class (e.g., terpenoids, flavonoids, and glucosinolates). Furthermore, the authors discuss strategies for the identification of metabolites and highlight how these anti-nematode metabolites might be of use in crop protection (Desmedt et al.). In this sense, the spray of sugar beet with active enzymes as ascorbate oxidase (an enzyme catalyzing the oxidation of apoplastic ascorbic acid to dehydroascorbic acid) induced plant defenses in a systemic way, resulting in a reduction in the number of *H. schachtii* infecting the plant (Singh et al.). This resistance induced by ascorbate oxidase is partially dependent on the jasmonic acid, ethylene, and salicylic acid pathways (Singh et al.). Another line of research is to induce the plant's defense by using microorganisms as a part of the PPNs management. Wang et al. demonstrated enhanced soybean resistance against SCN by using *Sinorhizobium freedi* strain Sneb183 in an iTRAQ-based proteomic analysis. The results suggested that the *S. fredii* Sneb183 may have a role in inducing isoflavonoid biosynthesis, thereby resulting in enhanced resistance to SCN infection in soybean. In some cases, the plant inoculation by a microorganism or derived compounds produced by microorganisms could induce a priming response by the plant to specific PPNs. Priming of plants by an inducer results in a faster and stronger response of the plant defense upon pathogen attack and could become an environmentally friendly strategy for plant protection (Borges et al., 2014; Conrath et al., 2015). The primed state could be even transgenerationally transmitted (Ramírez-Carrasco et al., 2017). Adss et al. studied the priming induction of soybean cv. Primus by the root application of the compound *N*-3-oxo-tetradecanoyl-*L*-homoserine lactone and the inoculation of the rhizobacterium producer of this compound (*Ensifer meliloti* strain ExpR+) against the root-lesion nematode, *Pratylenchus penetrans*. The results showed reduced root invasion compared to non-primed plants. Primed activated defense upon nematode invasion was indicated by increased production of the phytoalexin glyceollin by the soybean plant, while the preceding root application of the priming agent did not induce a direct defense response (Adss et al.). Compounds derived from microorganisms could also have a detrimental role in nematode infection and could be used for the development of new drugs. Prodoginines are red-colored tripyrrolic compounds produced by many bacterial species (Williamson et al., 2006). Habash et al. studied prodoginines and derivates against nematodes (*C. elegans* and *H. schachtii*) and plant pathogenic fungi (*Phoma lingam* and *Sclerotinia sclerotiorum*). *Caenorhabditis elegans* was affected by prodigiosin and the derivatives in a concentration-dependent manner while this effect was only detected for prodigiosin only for *H. schachtii* (Habash et al.). All compounds were found to be active against the plant pathogenic fungi studied (Habash et al.). Many of the advances in the development of environmentally benign treatments commented before are also applied in RKNs, and this has been reviewed by Forghani and Hajihassani in this Research Topic.

The ecological knowledge and requirements to activate the infective stage in a plant pathogen are essential points to design strategies for their control. In this sense, the hatching of CNs is finely tuned by the presence of the plant host in the majority of the species (Clarke and Perry, 1977). Ngala et al. studied the *in situ* hatching of encysted eggs of *G. pallida, H. carotae*, and *H. schachtii* after the application of root exudates at varying soil depths. The results showed that *G. pallida* hatching depends on soil moisture and effective exposure to root exudates, and to a lesser extent on exudate concentration. *Heterodera carotae* showed over 75% hatching induced by root exudate irrespective of the concentration, with better hatch induction at 20 cm than 10 cm soil depth, while *H. schachtii* mainly depends on the soil moisture level at constant temperature and is not influenced by the type or concentration of root exudates.

How plants and nematodes interact with each other is of great importance. Several systems have been studied in this Research Topic. RKNs infection of roots reactivates the cell cycle with a controlled balance of mitotic and endocycle programs (de Almeida Engler and Gheysen, 2013). Cabral et al. studied the role of the Armadillo BTB Protein ABAP1 in the DNA replication and transcription of nematode-induced galls by *Meloidogyne* spp. This study suggests that *ABAP1* is an essential component for cell cycle regulation throughout gall development and is required for feeding site homeostasis. Using the same group of nematodes, Truong et al. demonstrated that the *M. incognita* nuclear effector MiEFF1 interacts with *Arabidopsis* cytosolic glyceraldehyde-3-phosphate dehydrogenases during nematode parasitism. Plant resistance and susceptibility from RKN is studied using transcriptomics in several plant-nematode systems in this Research Topic as *M. incognita*-sweet potato (*Ipomoea batatas*) (Lee et al.), *M. incognita*-cucumber (*Cucumis sativus* and *C. metuliferus*) (Li et al.), and *M. arenaria*-*Solanum torvum* (Sato et al.). Zheng et al. reviewed in detail the molecular and cellular mechanisms involved in host-specific resistance to cyst nematodes in crops. This review explains in detail the knowledge of molecular and cellular mechanisms involved in the recognition of cyst nematodes, the activation of defense signaling, and resistance response types mediated by R genes or quantitative trait loci.

Pine wilt disease is caused by the pinewood nematode (PWN), *Bursaphelenchus xylophilus*, and constitutes one of the most important diseases in world forestry. This nematode is native to North America where it is not a pathogen to the endemic pine trees, but it has spread to the far East and some parts of Europe by world trading, causing serious damage to the local susceptive trees (Vicente et al., 2012). In this sense, it is a smart strategy to compare interactions between *Pinus* species with different sensitivities and nematodes with different virulence. Silva et al. studied the comparative analysis of PWN secretome under *P. pinaster* (high-susceptible host) and *P. pinea* (low-susceptible host) stimuli using a proteomic approach. In total, 22 proteins were found to be increased in the PWN secretome under the *P. pinaster* stimulus and 501 proteins increased under the *P. pinea* stimulus, with different protein activities in each host. In addition, the nematode secretome was also conducted by Shinya et al. to identify the virulence determinants of different isolates of PWN with distinct virulence levels. Highly secreted proteins from virulent *B. xylophilus* were selected as candidate virulent factors, and functions of two of them were clearly shown: a glycoside hydrolase family 30 (Bx-GH30) induce plant cell death, and one C1A family cysteine peptidase (Bx-CAT2) was involved in nematode feeding (Shinya et al.).

The editors of this Research Topic thank all authors who contribute and hope that this Research Topic helps the readers to understand and put in a higher view the problems caused by PPNs and their possible solutions inthe future.

## Author Contributions

JEP-R: conceptualization and writing—original draft. JEP-R, KH, SS, and CSLV: writing—review & editing.

## Conflict of Interest

The authors declare that the research was conducted in the absence of any commercial or financial relationships that could be construed as a potential conflict of interest.

## Publisher's Note

All claims expressed in this article are solely those of the authors and do not necessarily represent those of their affiliated organizations, or those of the publisher, the editors and the reviewers. Any product that may be evaluated in this article, or claim that may be made by its manufacturer, is not guaranteed or endorsed by the publisher.
